# Changes in tobacco and alcohol consumption during the COVID-19 pandemic in India: a propensity score matching approach

**DOI:** 10.1136/bmjgh-2023-013295

**Published:** 2024-11-24

**Authors:** Amit Summan, Ramanan Laxminarayan

**Affiliations:** 1 One Health Trust, Washington, Washington, USA; 2 One Health Trust, Bengaluru, Karnataka, India

**Keywords:** COVID-19, Public Health, Health policy, Control strategies, Prevention strategies

## Abstract

**Objectives:**

The COVID-19 pandemic may have influenced alcohol and tobacco consumption in low-income and middle-income countries, yet the effects are relatively unknown. In this study, we estimated the medium-term effects of the pandemic on tobacco and alcohol consumption in India.

**Methods:**

We used data from the fifth round of the National Family Health Survey 2019–2021, a nationally representative survey conducted between June 2019 and April 2021. We employed propensity score matching to evaluate the change in tobacco and alcohol consumption patterns by exploiting the gap in survey activities due to the pandemic lockdown—no data collection took place from April to October 2020. Individuals surveyed after the lockdown were considered COVID-19-affected, while those surveyed before were considered as unaffected.

**Results:**

The tobacco use rate was 1.4% lower and alcohol consumption was 0.3% lower for COVID-19-affected individuals relative to non-affected individuals. By tobacco product, there was a 0.9%, 0.6% and 0.4% decrease in the use of smokeless tobacco, cigarettes and bidi, respectively. Recent initiation decreased by 2.3%, 1.6% and 1.4%, for cigarettes, smokeless tobacco and alcohol, respectively. Tobacco use declined to a greater extent in low-wealth and rural populations, and in male and older subsamples. Alcohol use decreased in urban households, and among male and young subsamples, relative to their counterparts. Secondhand smoke exposure decreased by 4.6%.

**Conclusion:**

Tobacco and alcohol consumption, including recent initiation, decreased during the pandemic in India. Varying effects by subgroups suggest the need for targeted future control policies that support cessation and limit consumption.

**WHAT IS ALREADY KNOWN ON THIS TOPIC**

- Previous research on changes in tobacco and alcohol consumption during the COVID-19 pandemic is largely based in developed countries.
- Studies in India primarily consist of cross-sectional telephone and internet surveys conducted during the early stages of the pandemic using small sample sizes.

**WHAT THIS STUDY ADDS**

- This is the first study to examine the changes in tobacco and alcohol consumption during the COVID-19 pandemic in India, using large-scale household data.
- Disaggregated analysis provides insights into heterogeneous effects of non-pharmaceutical interventions on consumption that is deleterious to health.

**HOW THIS STUDY MIGHT AFFECT RESEARCH, PRACTICE OR POLICY**

- The findings from this study provide insights into the behaviour of individuals vis-à-vis substance use during future emergencies, specifically pandemics, in India.
- Changes in consumption patterns may vary by socioeconomic and demographic groups. Groups that are relatively likely to increase consumption should be targeted with modified control measures and those likely to attempt consumption reduction should be supported with cessation services.
- The drivers of changes in consumption in specific socioeconomic groups should be investigated in future research.

## Introduction

 The COVID-19 pandemic was characterised by widespread lockdowns in many parts of the world, particularly in India, where a strict lockdown was implemented on 25 March 2020, with mobility restrictions and curfews. During the early stages of the COVID-19 pandemic, there was concern that the policy response could increase the consumption of tobacco and alcohol, due to social isolation; stress or depression due to increased childcare burden and income loss; and boredom during lockdowns, among other factors.^1 2^ On the other hand, policies could have led to a decrease in tobacco and alcohol consumption due to reduced access, limited opportunities for social consumption, fear of COVID-19-related health outcomes associated with substance use, increased prices due to supply chain issues and reduced discretionary spending.^1 2^ Evidence from previous public health and economic crises suggested two opposing potential effects—increased consumption, particularly among men, due to pandemic-induced stressors and decreased consumption due to lack of access and budget constraints.^3 4^ The increased consumption can be explained by the ‘stress-response dampening theory’ and ‘self-medication theory’, particularly for alcohol consumption.^4^

India implemented one of the strictest lockdowns globally during the pandemic. The nationwide lockdown was accompanied by a national ban on alcohol from March to May 2020 for a 6-week period, after which states were allowed to determine their own rules for alcohol consumption. States had varied responses including alcohol being listed as an essential good, limited consumption being allowed in some states and complete prohibition in others. Moreover, policies changed during different stages of the pandemic.^5^ For tobacco, there was a ban on public use in many states and on spitting, ostensibly to limit COVID-19 transmission. The ban on spitting was relevant to consumption of smokeless (oral) tobacco.^6^

This study focuses on the overall effect of the pandemic and lockdowns on tobacco and alcohol consumption rates in India. Previous analyses have generally focused on higher-income countries.^7^,^10^ A meta-analysis of tobacco smoking during the prevaccination phase of the pandemic found substantial heterogeneity across countries, with an average decrease in smoking prevalence.^10^ One India-based study was included that had a ‘high risk’ of bias; the study conducted an online survey of 994 participants using snowball sampling.^11^ A systematic review of studies outside India found high variation in change in alcohol consumption across countries, but generally an increase in consumption and corresponding alcohol-related treatment needs.^7^ Studies from India have been cross-sectional in nature and included small and limited samples (<1000 individuals).^12^,^15^

In 2019, tobacco and alcohol caused an estimated 220 million and 93 million disability-adjusted life-years (DALYs) in India, respectively, with tobacco ranking as the third-largest contributor to DALYs among all risk factors.^16^ In 2017–2018, the economic costs (medical and non-medical expenditure, indirect morbidity costs, and indirect premature mortality cost) due to tobacco were US$27.5 billion.^17^ The cost of alcohol to the Indian economy was estimated at 1.45% annually between 2011 and 2050.^18^ According to the National Family Health Survey (NFHS) 2019–2021, which covered both pre-COVID-19 and post-COVID-19 periods, approximately 38.0% and 18.8% of men and 8.9% and 1.3% of women use tobacco and alcohol, respectively.^19^ Prepandemic projections in India suggested a trend towards decreasing tobacco consumption due to engaged tobacco control efforts^20^ by the government and non-governmental actors, and increasing alcohol consumption due to changing preferences in India.^21^

NFHS data on postpandemic consumption of alcohol and tobacco have only recently become available and enable an assessment of the impact of pandemics and countermeasures on alcohol and tobacco consumption patterns using a robust methodological approach. This study analysed the first large-scale nationally representative post-COVID-19 health survey in India to estimate the changes in tobacco and alcohol consumption before and after the pandemic. These findings are crucial for shaping tobacco and alcohol control policies and developing a ‘preparedness plan’ for future public health emergencies to avert a potentially substantial disease burden.

## Methods

### Data

We used data from the fifth round of the NFHS (NFHS-5),^19 22^ a cross-sectional, nationally representative health survey conducted between 2019 and 2021. The first phase of the survey was conducted between June 2019 and January 2020, covering 22 states and union territories (UTs) and the second phase was conducted between January 2020 and April 2021, covering 14 states and 3 UTs. No survey activities took place between April 2020 and October 2020 due to COVID-19-related lockdowns. The NFHS-5 sample followed a two-stage stratified sampling approach. The 2011 census was used as the sampling frame to select primary sampling units (PSUs). In rural areas, PSUs corresponded to villages, while in urban areas, they were Census Enumeration Blocks. Data were collected from 636 699 households in 707 districts.

For our analysis, the impact of the COVID-19 pandemic began on the date of the national lockdown, which was 25 March 2020. We considered individuals surveyed by NFHS-5 before COVID-19 (June 2019–March 2020) as the COVID-19-unaffected group and those surveyed after the COVID-19 lockdown (November 2020–April 2021) as the COVID-19-affected group. Therefore, in our study, ‘COVID-19-affected’ does not refer to whether individuals were infected with, or exposed to COVID-19, but only indicates survey timing.

We examined 13 outcomes related to consumption of tobacco: consumption of (1) any tobacco, (2) cigarettes, (3) bidis and (4) smokeless tobacco. We also examined the number of (5) cigarettes and (6) bidis, consumed in the last 24 hours and if the individual was a daily consumer of (7) cigarettes, (8) bidis and (9) smokeless tobacco. Lastly, we looked at recent initiation of (11) cigarettes, (12) bidis and (13) smokeless tobacco and (14) secondhand smoke exposure. For alcohol, we looked at three outcomes: (1) alcohol consumption, (2) daily alcohol consumption and (3) recent alcohol initiation. All outcome variables were binary, except for outcomes 6 and 7. Recent consumption was defined as initiation within the last 8 months. The gap between survey activities is eight months; therefore, any ‘recent’ initiator in the second round would have begun consumption only after the lockdown occurred.

### Empirical approach

Propensity score matching (PSM) was employed to estimate the effect of the COVID-19 pandemic on tobacco and alcohol consumption. Individual characteristics—including socioeconomic and demographic factors—may differ systematically between intervention and control groups in observational data. A correlation in these differences with outcome indicators would permit a comparison of unadjusted group means or an estimation of the association between intervention status and outcomes to be biased when analysed using ordinary least squares. PSM is a quasi-experimental approach used to analyse the effects of interventions in these types of non-experimental data.^23^,^26^ Individuals in the COVID-19-affected group were solely from NFHS-5 phase 2 states, as compared with the unaffected group, which had individuals from both phase 1 and phase 2 states. Systematic differences between states, such as standard of living or health status, could influence differences in changes in consumption patterns if they are not adequately controlled. For example, if states surveyed in the second phase were wealthier or had populations with healthier lifestyles, a negative least squares estimate of the association between pandemic ‘exposure’ and consumption outcomes may be biased downward.

PSM allows for the comparison of COVID-19-affected and unaffected populations that have similar observed characteristics. Essentially, each COVID-19-affected individual is matched with one who was not affected but had a similar probability of being affected based on observable characteristics. Through this matching process, differences in outcomes between COVID-19-affected and unaffected populations would be attributable to the pandemic under the assumption that all unobservable factors are randomly distributed between the treatment and control groups. The average difference—average treatment effect on the treated—in the outcome of interest is taken between these matched groups to determine the effect of the pandemic.^23^,^26^

We used a probit model to regress the binary indicator of exposure to the COVID-19 pandemic on the following covariates: region, locality (urban or rural), wealth quintile, religion, caste, age, sex, marital status, education level, body mass index (underweight, normal, overweight or obese) and level of fruit consumption (never, occasional, weekly or daily). We include matching variables that may be associated with survey timing (inclusion within COVID-19-affected or unaffected group) and the outcome variables of interest.^27^ These socioeconomic, demographic and lifestyle characteristics have been associated with tobacco and alcohol use in India in previous studies.^28 29^ To account for differences in underlying tobacco and alcohol consumption preferences of states and state-level policies towards alcohol and tobacco, we included four additional matching variables: (1) tobacco use among men, (2) tobacco use among women, (3) alcohol use among men and (4) alcohol use among women, using data from the fourth round of NFHS, conducted between 2015 and 2016. In NFHS-5, Daman & Diu and Dadra & Nagar Haveli were merged into one UT, for which we calculated a population-weighted average for each of these four variables. Additionally, Jammu and Kashmir (J&K) was divided into two UTs, J&K and Ladakh; therefore, in our dataset, these UTs had the same values for these variables.

The predicted probability or ‘propensity score’ from this regression was used to match COVID-19-affected and unaffected individuals. One-to-one, nearest-neighbour matching with replacement with common support was employed and heteroskedastic-consistent analytical standard errors were used from the nearest neighbour.^30^ Lastly, we conducted subsample analysis—outcomes were analysed by sex, income (top 3 vs bottom 2 wealth quintiles), locality and age group (under 21 years or 21 years and above).

The quality of PSM was analysed using three statistical indicators: (1) the change in the mean and median percentage bias—percentage of the square root of the average or median of the sample variance of the groups—across matching variables before and after matching, (2) the p value of the likelihood ratio test of joint significance of matching variables on the propensity score and (3) the pseudo R^2^ of the PSM model. For the latter two, the subsample matched observations from both groups are used to conduct first-stage PSM again, which provides an R^2^ value. A lower pseudo R^2^ or higher p value after matching indicates a reduction in systematic differences across variables. Stata V.14.2 was used to conduct all analyses and results are presented at 95% statistical significance.

### Patient and public involvement

This research was conducted without patient involvement. Patients were not invited to comment on the study design and were not consulted to develop patient-relevant outcomes or interpret the results. Patients were not invited to contribute to the writing or editing of this document for readability or accuracy.

## Results

### Summary statistics

Table 1 presents the differences in key background demographic, socioeconomic and health characteristics between COVID-19-affected and unaffected individuals. Due to survey timing, relative to the non-affected groups, there were larger COVID-19-affected populations in the Central region (47% vs 12%, p<0.01) and fewer in the Northeast (5% vs 18%, p<0.01) and West (0% vs 15%, p<0.01) regions. The largest difference among other variables was the greater proportion of COVID-19-affected Hindu households (82% vs 72%, p<0.01). Other discernible patterns in differences between intervention and control populations included lower fruit consumption (9% vs 11%, p<0.01).

**Table 1:** Differences in key background characteristics between COVID-19-affected and non-affected populations

	Intervention mean	Intervention SD	Control mean	Control SD	Difference	P value
Consume tobacco	0.09	0.28	0.12	0.32	−0.03**	0.00
Consume alcohol	0.05	0.22	0.05	0.21	0**	0.00
Region						
North	0.23	0.42	0.19	0.39	0.04**	0.00
Central	0.47	0.50	0.12	0.33	0.35**	0.00
East	0.16	0.36	0.16	0.37	−0.01**	0.00
Northeast	0.05	0.22	0.18	0.39	−0.13**	0.00
West	0.00	0.00	0.15	0.35	−0.15**	0.00
South	0.09	0.28	0.19	0.39	−0.1**	0.00
Rural	0.09	0.28	0.19	0.39	−0.1**	0.00
Wealth quintile						
1	0.23	0.42	0.19	0.39	0.04**	0.00
2	0.20	0.40	0.23	0.42	−0.03**	0.00
3	0.18	0.39	0.22	0.42	−0.04**	0.00
4	0.18	0.38	0.20	0.40	−0.02**	0.00
5	0.20	0.40	0.15	0.36	0.05**	0.00
Religion						
Hindu	0.82	0.38	0.72	0.45	0.09**	0.00
Muslim	0.08	0.28	0.14	0.35	−0.06**	0.00
Sikh	0.03	0.18	0.09	0.28	−0.05**	0.00
Christian	0.03	0.17	0.02	0.13	0.01**	0.00
Other	0.03	0.17	0.02	0.15	0.01**	0.00
Caste						
SC	0.21	0.41	0.19	0.39	0.02**	0.00
ST	0.19	0.39	0.18	0.39	0.01**	0.00
OBC	0.41	0.49	0.37	0.48	0.04**	0.00
Other	0.19	0.39	0.26	0.44	−0.08**	0.00
Married	0.69	0.46	0.70	0.46	−0.01**	0.00
Age	30.34	10.08	30.75	10.07	−0.4**	0.00
Female	0.88	0.33	0.88	0.33	0.00	0.87
Education						
Primary	0.12	0.32	0.12	0.32	0*	0.04
Secondary	0.50	0.50	0.53	0.50	−0.03**	0.00
Higher	0.16	0.36	0.14	0.35	0.02**	0.00
Body mass index						
Underweight	0.15	0.36	0.15	0.36	0**	0.00
Normal	0.50	0.50	0.51	0.50	−0.02**	0.00
Overweight	0.14	0.34	0.14	0.35	−0.01**	0.00
Obese	0.05	0.22	0.04	0.21	0**	0.00
Fruit consumption						
Never	0.01	0.10	0.01	0.12	0**	0.00
Occasionally	0.46	0.50	0.42	0.49	0.04**	0.00
Weekly	0.31	0.46	0.33	0.47	−0.02**	0.00
Daily	0.09	0.29	0.11	0.31	−0.02**	0.00
Sample size	259 662		566 292			

Intervention population is defined as those surveyed after the first COVID-19 lockdown.

Source: Authors’ estimates.

*p<0.05, **p<0.01.

OBC, Other Backward Caste; SC, Scheduled Caste; ST, Scheduled Tribe.

### Effects of COVID-19 on tobacco and alcohol consumption

The results of the propensity score model are presented in table 2. Almost all measures of tobacco and alcohol consumption decreased during the pandemic, with alcohol consumption showing relatively modest changes. The tobacco smoking rate was 1.4% (95% CI 1.1% to 1.7%, p<0.01) lower and alcohol consumption was 0.3% (95% CI 0.1% to 0.5%, p<0.05) lower for COVID-19-affected individuals versus non-affected individuals. For tobacco, the greatest decreases were for smokeless tobacco (0.9%), followed by cigarettes (0.6%) and then bidis (0.4%). The number of bidis smoked in the previous 24 hours decreased by 0.7 from an average of 8.7 bidis smoked prepandemic, while no significant effect was found in change in consumption of cigarettes. There were decreases in the prevalence of daily use of smokeless tobacco (0.8%), bidi (0.3%) and cigarettes (0.2%) and in the recent initiation of cigarettes (2.3%), smokeless tobacco (1.6%) and alcohol (1.4%). Exposure to secondhand smoke decreased by 4.6% (95% CI 4.0% to 5.1%, p<0.01).

**Table 2:** Propensity score results of the effect of COVID-19 on alcohol and tobacco consumption

Exposure	Outcome	Average treatment effect	P value	Lower bound	Upper bound	Sample size
Tobacco	Consume any form of tobacco (%)	−1.4	0.000	−1.7	−1.1	742 525
	Consume cigarettes (%)	−0.6	0.000	−0.7	−0.5	742 525
	Consume bidi (%)	−0.4	0.000	−0.5	−0.3	742 525
	Consume smokeless tobacco (%)	−0.9	0.000	−1.2	−0.6	742 525
	Number of cigarettes consumed in previous 24 hours	0.1	0.793	−0.4	0.5	12 976
	Number of bidis consumed in previous 24 hours	−0.7	0.020	−1.2	−0.1	11 596
	Daily cigarette consumer (%)	−0.2	0.000	−0.2	−0.1	742 525
	Daily bidi consumer (%)	−0.3	0.000	−0.5	−0.2	742 525
	Daily smokeless tobacco consumer (%)	−0.8	0.000	−1.0	−0.5	742 525
	Recently initiated cigarettes (%)	−2.3	0.022	−4.3	−0.3	12 606
	Recently initiated bidis (%)	−0.6	0.442	−2.0	0.9	11 515
	Recently initiated smokeless tobacco (%)	−1.6	0.000	−2.3	−0.8	59 865
	Exposed to secondhand smoke (%)	−4.6	0.000	−5.1	−4.0	742 525
Alcohol	Consume alcohol (%)	−0.3	0.004	−0.5	−0.1	742 525
	Daily alcohol consumer (%)	0.0	0.414	−0.1	0.0	742 525
	Recently initiated of alcohol (%)	−1.4	0.002	−2.3	−0.5	32 273

All outcome variables are binary (no=0, yes=1) except for number of cigarettes/bidis consumed in the previous 24-hour period. Recent initiation refers to initiation within the previous eight months.

Source: Authors’ estimates.

Online supplemental table A1 shows that PSM decreased the differences across COVID-19-affected and non-affected groups in the intervention and control group matching variables. The mean and median percentage differences in matching variables decreased substantially across the two groups. Also, the pseudo R^2^ was lower, and the p value of the joint significance test was higher and insignificant in the matched sample.

### Subsample analysis

Results by wealth, location, age and gender subsample are presented in online supplemental table B1. Tobacco use declined to a greater extent for users in low-wealth and rural households, as well as for male and older populations relative to their counterparts. Alcohol use decreased in high-wealth and urban households, and for younger and male subsamples, while no decrease was found for their counterparts; however, these latter groups—low-wealth, rural, female and older populations—did have decreases in recent initiation. The largest differential across wealth groups was in the relatively larger decreases in smokeless tobacco and bidi consumption in low-wealth versus high-wealth households. For rural households the decreases were largest in bidi consumption relative to urban households, where no change was seen in the latter. Males had substantially greater decreases in tobacco consumption relative to females, 4.0% vs 1.1%. However, the recent initiation of alcohol decreased to a greater extent for females. Online supplemental tables A2–A5 show the results from the quality of matching tests—there were reductions in systematic differences between intervention and control groups on matching variables after PSM.

## Discussion

The policies implemented to curb COVID-19 transmission had wide-ranging impacts on daily life. While lockdown policies curbed the transmission and spread of the virus, they also restricted mobility, caused social isolation, and impacted economic activity. Our analysis found that almost all measures of tobacco and alcohol consumption decreased during the pandemic, with the largest reductions seen in new users. The tobacco smoking rate was 1.4% lower and alcohol consumption was 0.3% lower for COVID-19-affected individuals compared with non-affected individuals. There were decreases in the prevalence of daily use of smokeless tobacco, bidis and cigarettes. Additionally, recent initiation of bidi, smokeless tobacco and alcohol decreased substantially more than overall consumption. Our estimates suggest that both alcohol and tobacco consumption decreased beyond expected consumption changes according to prepandemic trend levels—from 2005–2006 to 2015–2016, male tobacco and alcohol consumption decreased by 12.5% and 2.7%, while we found decreases of 4.2% and 2.5%, respectively.^31^

The magnitude of decreases varied across socioeconomic and demographic groups. Tobacco use decreased to a greater extent in low-wealth and rural subsamples, and male and older populations relative to their counterparts. For alcohol, decreases were larger for users in urban and high-wealth households, and for male and young subsamples. Low-wealth households did not have significant decreases in alcohol consumption. Males experienced substantially greater decreases in tobacco and alcohol consumption relative to females. However, the recent initiation of alcohol decreased to a greater extent for females.

There are several possible explanations for these findings. Evidence from previous public health and economic crises suggests two possible pathways: increased consumption, especially in men, due to pandemic-induced stressors and decreased consumption because of lack of access due to supply chain disruptions, reduced incomes and other barriers such as curfews.^3^ Additionally, opportunities for consumption of alcohol in social settings were limited during lockdown periods, especially for younger populations.^32 33^ A 2015 systematic review of the effect of economic crises on alcohol consumption found an increase in alcohol consumption in men, but not women. However, none of the reviewed studies included India. A survey conducted in India during the pandemic found men were more likely to resort to maladaptive coping practices such as alcohol while females were more likely to reach out to existing support networks, potentially due to gender roles within Indian cultures.^34^ We found that alcohol consumption in men decreased to a greater extent than in women during the pandemic, suggesting a strong role for lack of access or budget constraints during the pandemic. This may also be due to excise tax increases on alcohol to respond to COVID-19 during this period; for example, Delhi had a 70% temporary ‘corona’ tax for May 2020 which was repealed in June 2020.^35^ A survey conducted from July to August 2020 found that tobacco users in India who were aware of the effects of tobacco on COVID-19 were more likely to either quit use or attempt quitting more frequently during the lockdown.^36^ This may explain why older populations had higher decreases in tobacco consumption, as they were at more risk to suffer from more severe COVID-19 outcomes if infected. Lastly, a telephone survey conducted in May 2020 found that 51% of participants in a tobacco cessation programme that quit reported the pandemic impacted their decision to quit, while 27% and 45% of all participants indicated that price increases and lack of tobacco availability were responsible for their decision.^6^

It is unclear what may have caused greater decreases in tobacco for low-wealth households, but larger decreases in alcohol for high-wealth households. In one US study, while tobacco use decreased during the pandemic overall it increased in the higher socioeconomic status groups.^2^ These effects may be related to overall budgetary considerations and also relative preferences which can be studied further.

An analysis of data from the Centre for Monitoring Indian Economy–Consumer Pyramids Household Survey found an increase in alcohol consumption expenditure as a share of total expenditure during the lockdown period peaking in April 2020 relative to a prepandemic period starting from July 2019, however, expenditure reduced to prepandemic levels post this period.^37^ The spike may represent an increase in alcohol consumption or increased prices of alcohol due to temporary taxes or reduced availability; however, we were unable to assess changes in actual consumption from this analysis.

Our results and broader findings from related studies^7^ have several policy implications for alcohol and tobacco control. Broadly, the varying effects of socioeconomic and demographic groups highlight potential strategies that should be highly targeted across groups for tobacco and alcohol control policy in the future. Specifically, first, it is critical to provide strong messaging across different forms of media on the effects of comorbidities on pandemic outcomes. The COVID-19 pandemic presented a unique opportunity to target consumption by highlighting the adverse effects of tobacco and alcohol consumption for COVID-19 outcomes, which showed to have an effect on consumption.^36^ Second, as the pandemic may have spurred increased tobacco quit attempts, there should be expanded coverage of cessation aid services for individuals such as toll-free quit lines and m-cessation. An article, largely referencing news media, suggested the consequences of alcohol unavailability included withdrawal, increased black market activity and suicides from withdrawal symptoms.^5^ The treatment gap for alcohol use disorder is 82.7% globally with a greater gap in LMICs.^38^ When there is a sudden decrease in alcohol availability, expanded services for those who may face alcohol withdrawal symptoms should be considered. Third, monitoring of alcohol and tobacco consumption through surveys, sales records, health providers and addiction centres can all provide a better picture of consumption patterns throughout the pandemic to support real-time modifications to policies as the need arises.^39^ Lastly, the effects of broader tax policy should be considered. As alcohol taxation is still determined by individual states and UTs, the COVID-19 pandemic along with delayed funding transfers to states may have incentivised many states to prematurely increase access to alcohol to generate tax revenue despite social distancing concerns.^40^

There are important limitations to our study. First, our findings only identify the change in consumption of alcohol and tobacco for the survey period. The post-COVID-19 survey was conducted between November 2020 and March 2021. This was right after the first lockdown and before the second COVID-19 wave in India that began in late March 2021 due to mass gatherings in religious festivals and political rallies.^41^ It is possible that during future phases of the pandemic and postpandemic periods, different pathways—due to health concerns, affordability, accessibility and other policy responses—may change the trajectory of consumption. Our results only pertain to the period under consideration.

Second, although in our PSM analysis, we accounted for a wide range of potentially confounding factors, it is possible that there are unobserved characteristics varying systematically between the COVID-19-affected and unaffected groups. If these differences are correlated with the outcome variables, they may bias our estimates. Third, our analysis is based on self-reported data which may suffer from recall bias especially variables relating to intensity of consumption. However, the questions in the survey had been designed to limit such bias (eg, asking about consumption in the past 24 hours). Lastly, due to a lack of data, we were unable to determine the exact mechanisms through which changes in tobacco and alcohol consumption may have occurred. However, we have described potential pathways by reviewing past literature, and surveys conducted during COVID-19.

## Conclusion

The COVID-19 pandemic presented an unprecedented challenge to health systems and policymakers globally. Low-income and middle-income and resource-constrained countries were particularly challenged. It is important to document the secondary effects of the pandemic on risk factors for health, including tobacco and alcohol use. Our research showed that, in the short term, the pandemic did not lead to increased consumption of alcohol and tobacco. Future research can analyse if these changes in consumption will be sustained or revert to prepandemic levels.

## Supplementary material

10.1136/bmjgh-2023-013295 online supplemental file 1

10.1136/bmjgh-2023-013295 online supplemental file 2

## Data Availability

All data are publicly available and can be accessed through The DHS Program, https://dhsprogram.com/data/.
