# Supplementary material for: Changes in tobacco and alcohol consumption during the COVID-19 pandemic in India: a propensity score matching approach
Source: BMJ Glob Health. 2024 Nov 24;9(11):e013295. doi: 10.1136/bmjgh-2023-013295 (PMC11603733; doi:10.1136/bmjgh-2023-013295)
Supplement: online supplemental file 1 [file bmjgh-9-11-s001.pdf]

Table A1: Balancing between matched and control groups in main model

Outcome	Data	Pseudo R^2	$P>Chi^2$	Mean bias	Median bias
Consume any form of tobacco (%)	Matched	0.01	0.00	1.58	0.88
	Unmatched	0.21	0.00	10.27	5.21
Consume cigarettes (%)	Matched	0.01	0.00	1.58	0.88
	Unmatched	0.21	0.00	10.27	5.21
Consume bidi (%)	Matched	0.01	0.00	1.58	0.88
	Unmatched	0.21	0.00	10.27	5.21
Consume smokeless tobacco (%)	Matched	0.01	0.00	1.58	0.88
	Unmatched	0.21	0.00	10.27	5.21
Number of cigarettes consumed in previous 24 hours	Matched	0.04	0.00	4.05	3.68
	Unmatched	0.26	0.00	14.26	8.12
Number of bidis consumed in previous 24 hours	Matched	0.03	0.00	3.18	2.83
	Unmatched	0.24	0.00	14.34	7.95
Daily cigarette consumer (%)	Matched	0.01	0.00	1.58	0.88
	Unmatched	0.21	0.00	10.27	5.21
Daily bidi consumer (%)	Matched	0.01	0.00	1.58	0.88
	Unmatched	0.21	0.00	10.27	5.21
Daily smokeless tobacco consumer (%)	Matched	0.01	0.00	1.58	0.88
	Unmatched	0.21	0.00	10.27	5.21
Recently initiated cigarettes (%)	Matched	0.04	0.00	4.15	2.85
	Unmatched	0.26	0.00	14.04	7.71
Recently initiated bidis (%)	Matched	0.03	0.00	3.46	2.66
	Unmatched	0.23	0.00	14.34	8.85
Recently initiated smokeless tobacco (%)	Matched	0.01	0.00	1.90	1.28
	Unmatched	0.36	0.00	22.11	13.66
Exposed to second-hand smoke (%)	Matched	0.01	0.00	1.58	0.88
	Unmatched	0.21	0.00	10.27	5.21
Consume alcohol (%)	Matched	0.01	0.00	1.58	0.88
	Unmatched	0.21	0.00	10.27	5.21

Daily alcohol consumer (%)	Matched	0.01	0.00	1.58	0.88
	Unmatched	0.21	0.00	10.27	5.21
Recently initiated of alcohol (%)	Matched	0.01	0.00	3.14	2.72
	Unmatched	0.25	0.00	13.31	9.23

Note: Results from propensity score matching using one-to-one nearest neighbor matching and imposing common support.

Table A2: Balancing between matched and control groups in wealth group subsample analysis

Sample	Data	High-wealth				Low-wealth			
Outcome		Pseudo R^2	P>Chi^2	Mean bias	Median bias	Pseudo R^2	P>Chi^2	Mean bias	Median bias
Consume any form of tobacco (%)	Matched	0.02	0.00	2.29	1.60	0.01	0.00	1.83	1.41
	Unmatched	0.18	0.00	10.82	5.84	0.27	0.00	13.89	6.90
Consume cigarettes (%)	Matched	0.02	0.00	2.29	1.60	0.01	0.00	1.83	1.41
	Unmatched	0.18	0.00	10.82	5.84	0.27	0.00	13.89	6.90
Consume bidi (%)	Matched	0.02	0.00	2.29	1.60	0.01	0.00	1.83	1.41
	Unmatched	0.18	0.00	10.82	5.84	0.27	0.00	13.89	6.90
Consume smokeless tobacco (%)	Matched	0.02	0.00	2.29	1.60	0.01	0.00	1.83	1.41
	Unmatched	0.18	0.00	10.82	5.84	0.27	0.00	13.89	6.90
Number of cigarettes consumed in previous 24 hours	Matched	0.06	0.00	5.92	3.97	0.02	0.00	4.11	3.58
	Unmatched	0.24	0.00	16.12	10.21	0.31	0.00	16.43	7.84
Number of bidis consumed in previous 24 hours	Matched	0.03	0.00	3.80	3.29	0.02	0.00	4.25	3.35
	Unmatched	0.19	0.00	13.41	5.70	0.28	0.00	16.60	8.99
Daily cigarette consumer (%)	Matched	0.02	0.00	2.29	1.60	0.01	0.00	1.83	1.41
	Unmatched	0.18	0.00	10.82	5.84	0.27	0.00	13.89	6.90
Daily bidi consumer (%)	Matched	0.02	0.00	2.29	1.60	0.01	0.00	1.83	1.41
	Unmatched	0.18	0.00	10.82	5.84	0.27	0.00	13.89	6.90
Daily smokeless tobacco consumer (%)	Matched	0.02	0.00	2.29	1.60	0.01	0.00	1.83	1.41
	Unmatched	0.18	0.00	10.82	5.84	0.27	0.00	13.89	6.90
Recently initiated cigarettes (%)	Matched	0.05	0.00	6.01	4.85	0.03	0.00	3.82	2.71
	Unmatched	0.23	0.00	15.50	10.59	0.30	0.00	16.51	7.51
Recently initiated bidis (%)	Matched	0.00	0.00	0.00	0.00	0.02	0.00	3.25	2.71
	Unmatched	0.18	0.00	0.00	0.00	0.28	0.00	16.92	9.47
Recently initiated smokeless tobacco (%)	Matched	0.02	0.00	2.24	1.77	0.01	0.00	1.88	1.59
	Unmatched	0.35	0.00	24.96	8.10	0.37	0.00	23.59	15.95
Exposed to second-hand smoke (%)	Matched	0.02	0.00	2.29	1.60	0.01	0.00	1.83	1.41
	Unmatched	0.18	0.00	10.82	5.84	0.27	0.00	13.89	6.90

Consume alcohol (%)	Matched	0.02	0.00	2.29	1.60	0.01	0.00	1.83	1.41
	Unmatched	0.18	0.00	10.82	5.84	0.27	0.00	13.89	6.90
Daily alcohol consumer (%)	Matched	0.02	0.00	2.29	1.60	0.01	0.00	1.83	1.41
	Unmatched	0.18	0.00	10.82	5.84	0.27	0.00	13.89	6.90
Recently initiated of alcohol (%)	Matched	0.02	0.00	3.98	2.65	0.01	0.00	2.62	1.72
	Unmatched	0.17	0.00	9.90	5.46	0.32	0.00	16.32	11.41

Note: Results from propensity score matching using one-to-one nearest neighbor matching and imposing common support.

Table A3: Balancing between matched and control groups in sex group subsample analysis

Sample	Data	Male				Female			
Outcome		Pseudo R^2	P>Chi^2	Mean bias	Median bias	Pseudo R^2	P>Chi^2	Mean bias	Median bias
Consume any form of tobacco (%)	Matched	0.02	0.00	2.50	1.94	0.01	0.00	1.49	0.85
	Unmatched	0.21	0.00	11.72	5.19	0.21	0.00	10.85	5.60
Consume cigarettes (%)	Matched	0.02	0.00	2.50	1.94	0.01	0.00	1.49	0.85
	Unmatched	0.21	0.00	11.72	5.19	0.21	0.00	10.85	5.60
Consume bidi (%)	Matched	0.02	0.00	2.50	1.94	0.01	0.00	1.49	0.85
	Unmatched	0.21	0.00	11.72	5.19	0.21	0.00	10.85	5.60
Consume smokeless tobacco (%)	Matched	0.02	0.00	2.50	1.94	0.01	0.00	1.49	0.85
	Unmatched	0.21	0.00	11.72	5.19	0.21	0.00	10.85	5.60
Number of cigarettes consumed in previous 24 hours	Matched	0.05	0.00	5.02	3.71	0.15	0.00	10.76	8.08
	Unmatched	0.26	0.00	15.27	8.08	0.39	0.00	25.70	15.11
Number of bidis consumed in previous 24 hours	Matched	0.03	0.00	3.20	2.73	0.07	0.00	8.08	6.79
	Unmatched	0.23	0.00	15.34	8.18	0.34	0.00	15.83	10.21
Daily cigarette consumer (%)	Matched	0.02	0.00	2.50	1.94	0.01	0.00	1.49	0.85
	Unmatched	0.21	0.00	11.72	5.19	0.21	0.00	10.85	5.60
Daily bidi consumer (%)	Matched	0.02	0.00	2.50	1.94	0.01	0.00	1.49	0.85
	Unmatched	0.21	0.00	11.72	5.19	0.21	0.00	10.85	5.60
Daily smokeless tobacco consumer (%)	Matched	0.02	0.00	2.50	1.94	0.01	0.00	1.49	0.85
	Unmatched	0.21	0.00	11.72	5.19	0.21	0.00	10.85	5.60
Recently initiated cigarettes (%)	Matched	0.05	0.00	4.50	3.68	0.11	0.10	14.63	10.35
	Unmatched	0.25	0.00	15.07	6.88	0.36	0.00	23.75	18.71
Recently initiated bidis (%)	Matched	0.03	0.00	3.13	2.93	0.07	0.00	6.90	5.42
	Unmatched	0.23	0.00	15.49	8.86	0.34	0.00	15.64	10.38
Recently initiated smokeless tobacco (%)	Matched	0.01	0.00	2.44	1.46	0.01	0.00	2.19	1.71
	Unmatched	0.26	0.00	12.60	7.02	0.44	0.00	29.85	19.66
Exposed to second-hand smoke (%)	Matched	0.02	0.00	2.50	1.94	0.01	0.00	1.49	0.85
	Unmatched	0.21	0.00	11.72	5.19	0.21	0.00	10.85	5.60

Consume alcohol (%)	Matched	0.02	0.00	2.50	1.94	0.01	0.00	1.49	0.85
	Unmatched	0.21	0.00	11.72	5.19	0.21	0.00	10.85	5.60
Daily alcohol consumer (%)	Matched	0.02	0.00	2.50	1.94	0.01	0.00	1.49	0.85
	Unmatched	0.21	0.00	11.72	5.19	0.21	0.00	10.85	5.60
Recently initiated of alcohol (%)	Matched	0.02	0.00	4.29	3.64	0.03	0.00	4.51	3.24
	Unmatched	0.19	0.00	11.91	8.62	0.46	0.00	20.78	12.64

Note: Results from propensity score matching using one-to-one nearest neighbor matching and imposing common support.

Table A4: Balancing between matched and control groups in locality subsample analysis

Sample	Data	Urban				Rural			
Outcome		Pseudo R^2	$P>Chi^2$	Mean bias	Median bias	Pseudo R^2	$P>Chi^2$	Mean bias	Median bias
Consume any form of tobacco (%)	Matched	0.02	0.00	1.77	1.35	0.01	0.00	1.65	1.12
	Unmatched	0.20	0.00	11.40	6.05	0.23	0.00	11.42	6.98
Consume cigarettes (%)	Matched	0.02	0.00	1.77	1.35	0.01	0.00	1.65	1.12
	Unmatched	0.20	0.00	11.40	6.05	0.23	0.00	11.42	6.98
Consume bidi (%)	Matched	0.02	0.00	1.77	1.35	0.01	0.00	1.65	1.12
	Unmatched	0.20	0.00	11.40	6.05	0.23	0.00	11.42	6.98
Consume smokeless tobacco (%)	Matched	0.02	0.00	1.77	1.35	0.01	0.00	1.65	1.12
	Unmatched	0.20	0.00	11.40	6.05	0.23	0.00	11.42	6.98
Number of cigarettes consumed in previous 24 hours	Matched	0.08	0.00	5.31	5.25	0.04	0.00	3.56	2.46
	Unmatched	0.29	0.00	18.98	8.87	0.27	0.00	14.35	7.30
Number of bidis consumed in previous 24 hours	Matched	0.05	0.00	6.54	5.09	0.03	0.00	3.40	2.41
	Unmatched	0.28	0.00	18.23	9.19	0.24	0.00	14.69	9.64
Daily cigarette consumer (%)	Matched	0.02	0.00	1.77	1.35	0.01	0.00	1.65	1.12
	Unmatched	0.20	0.00	11.40	6.05	0.23	0.00	11.42	6.98
Daily bidi consumer (%)	Matched	0.02	0.00	1.77	1.35	0.01	0.00	1.65	1.12
	Unmatched	0.20	0.00	11.40	6.05	0.23	0.00	11.42	6.98
Daily smokeless tobacco consumer (%)	Matched	0.02	0.00	1.77	1.35	0.01	0.00	1.65	1.12
	Unmatched	0.20	0.00	11.40	6.05	0.23	0.00	11.42	6.98
Recently initiated cigarettes (%)	Matched	0.04	0.00	5.38	4.44	0.03	0.00	3.16	2.58
	Unmatched	0.29	0.00	18.14	7.64	0.26	0.00	14.39	8.34
Recently initiated bidis (%)	Matched	0.05	0.00	8.19	6.74	0.02	0.00	3.38	2.27
	Unmatched	0.28	0.00	17.14	9.19	0.24	0.00	14.77	8.63
Recently initiated smokeless tobacco (%)	Matched	0.02	0.00	2.78	2.05	0.01	0.00	1.78	1.32
	Unmatched	0.36	0.00	26.02	10.29	0.37	0.00	22.00	12.92
Exposed to second-hand smoke (%)	Matched	0.02	0.00	1.77	1.35	0.01	0.00	1.65	1.12
	Unmatched	0.20	0.00	11.40	6.05	0.23	0.00	11.42	6.98

Consume alcohol (%)	Matched	0.02	0.00	1.77	1.35	0.01	0.00	1.65	1.12
	Unmatched	0.20	0.00	11.40	6.05	0.23	0.00	11.42	6.98
Daily alcohol consumer (%)	Matched	0.02	0.00	1.77	1.35	0.01	0.00	1.65	1.12
	Unmatched	0.20	0.00	11.40	6.05	0.23	0.00	11.42	6.98
Recently initiated of alcohol (%)	Matched	0.04	0.00	3.99	2.79	0.01	0.00	2.39	1.83
	Unmatched	0.18	0.00	8.52	5.10	0.29	0.00	14.97	11.23

Note: Results from propensity score matching using one-to-one nearest neighbor matching and imposing common support.

Table A5: Balancing between matched and control groups in age group subsample analysis

Sample	Data	Young				Old			
Outcome		Pseudo R^2	P>Chi^2	Mean bias	Median bias	Pseudo R^2	P>Chi^2	Mean bias	Median bias
Consume any form of tobacco (%)	Matched	0.01	0.00	1.85	1.49	0.02	0.00	1.81	1.07
	Unmatched	0.21	0.00	10.20	5.31	0.21	0.00	10.43	5.14
Consume cigarettes (%)	Matched	0.01	0.00	1.85	1.49	0.02	0.00	1.81	1.07
	Unmatched	0.21	0.00	10.20	5.31	0.21	0.00	10.43	5.14
Consume bidi (%)	Matched	0.01	0.00	1.85	1.49	0.02	0.00	1.81	1.07
	Unmatched	0.21	0.00	10.20	5.31	0.21	0.00	10.43	5.14
Consume smokeless tobacco (%)	Matched	0.01	0.00	1.85	1.49	0.02	0.00	1.81	1.07
	Unmatched	0.21	0.00	10.20	5.31	0.21	0.00	10.43	5.14
Number of cigarettes consumed in previous 24 hours	Matched	0.03	0.00	4.84	4.16	0.05	0.00	4.49	4.20
	Unmatched	0.27	0.00	14.72	10.20	0.27	0.00	14.42	6.93
Number of bidis consumed in previous 24 hours	Matched	0.06	0.01	7.55	7.13	0.03	0.00	3.04	1.87
	Unmatched	0.34	0.00	17.12	8.85	0.23	0.00	14.27	7.25
Daily cigarette consumer (%)	Matched	0.01	0.00	1.85	1.49	0.02	0.00	1.81	1.07
	Unmatched	0.21	0.00	10.20	5.31	0.21	0.00	10.43	5.14
Daily bidi consumer (%)	Matched	0.01	0.00	1.85	1.49	0.02	0.00	1.81	1.07
	Unmatched	0.21	0.00	10.20	5.31	0.21	0.00	10.43	5.14
Daily smokeless tobacco consumer (%)	Matched	0.01	0.00	1.85	1.49	0.02	0.00	1.81	1.07
	Unmatched	0.21	0.00	10.20	5.31	0.21	0.00	10.43	5.14
Recently initiated cigarettes (%)	Matched	0.03	0.00	4.17	3.75	0.04	0.00	3.75	3.11
	Unmatched	0.26	0.00	14.52	9.87	0.26	0.00	14.28	5.96
Recently initiated bidis (%)	Matched	0.03	0.71	5.65	4.22	0.03	0.00	3.06	2.98
	Unmatched	0.31	0.00	15.77	6.56	0.23	0.00	14.32	7.85
Recently initiated smokeless tobacco (%)	Matched	0.01	0.00	2.41	1.59	0.01	0.00	2.21	1.41
	Unmatched	0.36	0.00	22.09	11.73	0.36	0.00	22.54	13.33
	Matched	0.01	0.00	1.85	1.49	0.02	0.00	1.81	1.07

Exposed to second-hand smoke (%)	Unmatched	0.21	0.00	10.20	5.31	0.21	0.00	10.43	5.14
Consume alcohol (%)	Matched	0.01	0.00	1.85	1.49	0.02	0.00	1.81	1.07
	Unmatched	0.21	0.00	10.20	5.31	0.21	0.00	10.43	5.14
Daily alcohol consumer (%)	Matched	0.01	0.00	1.85	1.49	0.02	0.00	1.81	1.07
	Unmatched	0.21	0.00	10.20	5.31	0.21	0.00	10.43	5.14
Recently initiated of alcohol (%)	Matched	0.02	0.00	3.09	2.09	0.01	0.00	3.11	2.31
	Unmatched	0.26	0.00	12.79	8.01	0.25	0.00	13.49	9.13

Note: Results from propensity score matching using one-to-one nearest neighbor matching and imposing common support.
